# Pediatric brain hydatid cyst about two cases: Case report

**DOI:** 10.1016/j.amsu.2022.103806

**Published:** 2022-05-18

**Authors:** Saad Hmada, Tarek Mesbahi, Abdelhamid Jehri, Abla Jouida, Abdessamad Naja, Naima Amenzoui, Abdelhakim Lakhdar

**Affiliations:** a Neurosurgery Department, University Hospital Center IBN Rochd, Casablanca, Morocco; b Pediatric Department, University Hospital Center Abderrahim Harouchi, Casablanca, Morocco

**Keywords:** Hydatid cyst, Brain, Echinococcus, Pediatric, Case report

## Abstract

Cerebral hydatid cyst is rare (2%), and mainly affects children. We report 2 cases aged 5 years. The clinical symptomatology was dominated by intracranial hypertension syndrome and motor deficit in both cases. One patient presented a generalized tonic-clonic seizure, the second one presented a left central facial palsy. The diagnosis was made in both cases by brain CT scan and one patient underwent brain MRI. A radiological workup to look for extra-cerebral localization was performed for all patients, which was normal. The treatment was surgical for both patients (D'ARANA-INIGUEZ hydro pulsion technique) with simple after-effects. The postoperative CT scan showed a residual cavity. All our patients were put under antiparasitic treatment with Albendazole.

## Highlights

• Two cases of children with motor deficit of progressive onset associated with intracranial hypertension.
• The clinical examination revealed a hemiplegic motor deficit the CT scan as well as the cerebral MRI were carried out which was in favor of the diagnosis.
• Confirmation with intraoperatively with the delivery of this one using technique of ARANA-INIGUEZ, and by the parasitological examination which objectified Echinococcus granulosus.

## 1 Introduction

Echinococcus granulosis is a parasite that lives in the intestines of animals, especially those in contact with humans, while its larvae cause hydatid cyst disease in humans, cattle, and sheep [1].

The eggs having penetrated the human body orally, hatch into larvae in the small intestine, which penetrate the intestinal wall and then hematogenous dissemination to reach several organs, most often liver (55–70%) or lungs (15–35%), 10% of the latter reach the systemic circulation and end up in other organs such as the brain. Primary intracranial cysts are very rare (1.7%), and 75% of patients are children.

## 2 Cases report

### 2.1 First case

Inas 5-years old girl with no pathological history and notion of contact with stray dogs. The history of the disease goes back to 6 months before her admission by an intracranial hypertension disorder made of headaches, vomiting in jet especially in the morning and a decrease of visual acuity. The picture was complicated 4 months later by tremors of the left hemi corpus during movements without any notion of convulsive seizures or consciousness disorder.

At the clinical examination, the patient was conscious 15/15, walking was possible with a slight enlargement of the sustentation polygon, muscle strength was 5/5 in all 4 limbs, coordination disorder of the left hemi corpus, without sensory disorders or attacks of the cranial pairs. The rest of the somatic examination was unremarkable.

The diagnostic hypotheses were in favor of an arachnoid cyst and a cerebral hydatid cyst but given the clinical and epidemiological context (contact with dogs and life in a rural environment), the diagnosis of cerebral hydatid cyst was more plausible. The search for other locations, particularly by abdominal ultrasound and chest X-ray, did not reveal any abnormalities (see Fig. 3, Fig. 4, Fig. 5, Fig. 6, Fig. 7, Fig. 8).

**Fig. 1:**
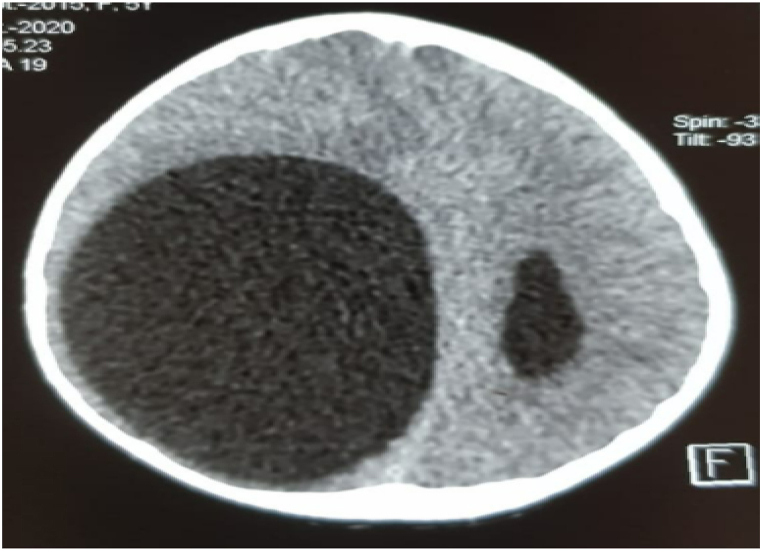
A brain CT scan was performed, showing a round hypodense intra-parenchymal lesion at the right fronto-temporal-parietal level with an important mass effect on the midline (Fig. 1).

**Fig. 2:**
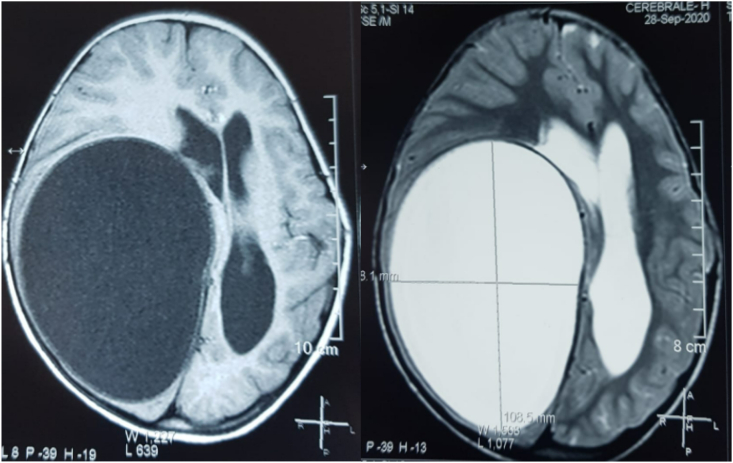
A cerebral MRI was performed showing a rounded hypointense lesion in T1, hyperintense in T2 at the right fronto-temporo-parietal level with an important mass effect and subfalcoral involvement in favor of a cerebral hydatid cyst (Fig. 2).

**Fig. 3:**
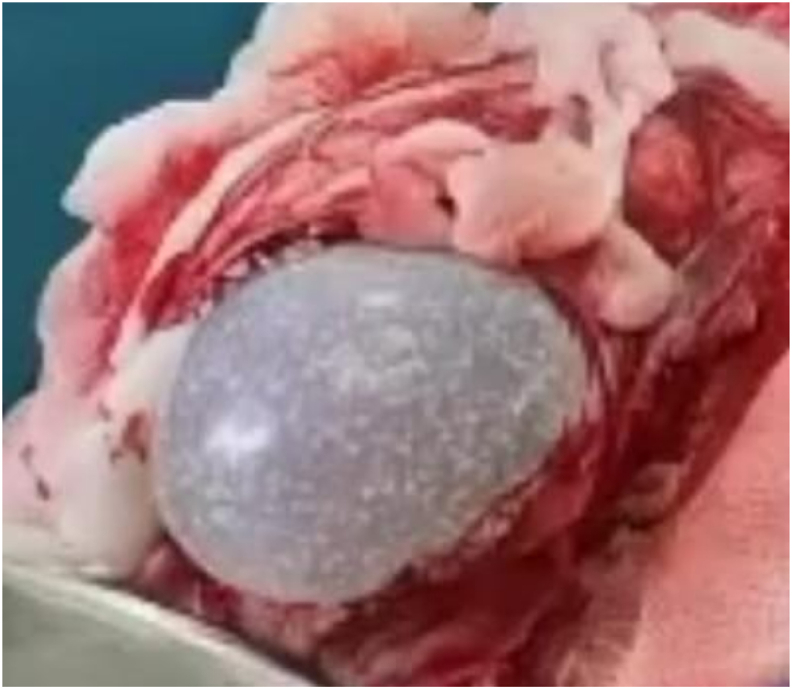
An intraoperative image showing the delivery of the hydatid cyst.

**Fig. 4:**
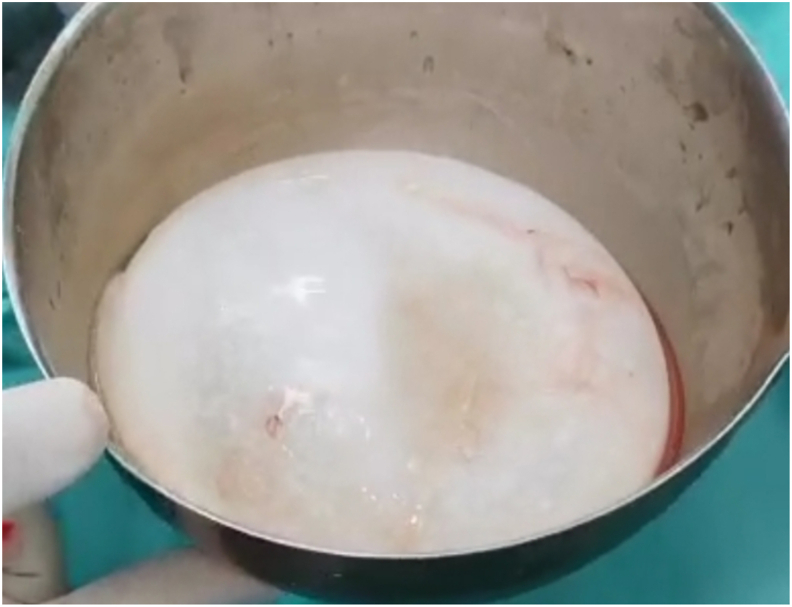
Image showing the complete removal of the hydatid cyst without its rupture.

**Fig. 5:**
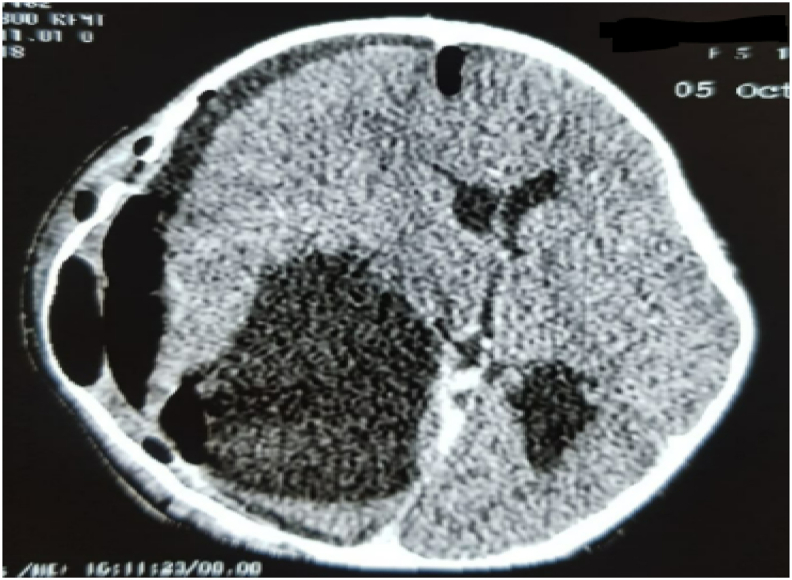
The control CT scan showing the total removal of the hydatid cyst.

**Fig. 6:**
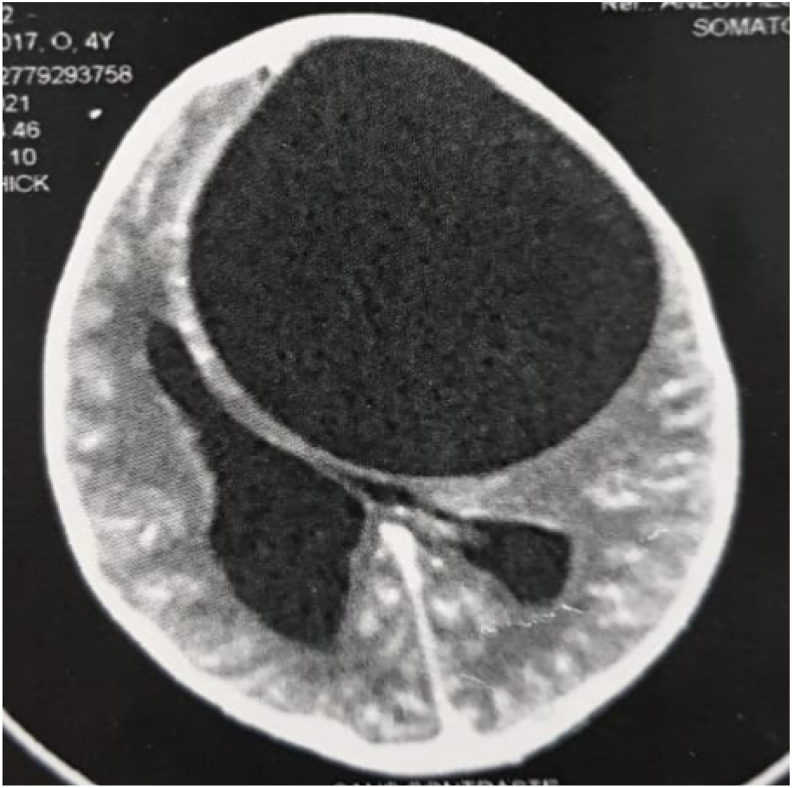
A brain CT scan was performed, showing a round hypodense intra-parenchymal lesion at the left fronto-parietal level with an important mass effect on the midline.

**Fig. 7:**
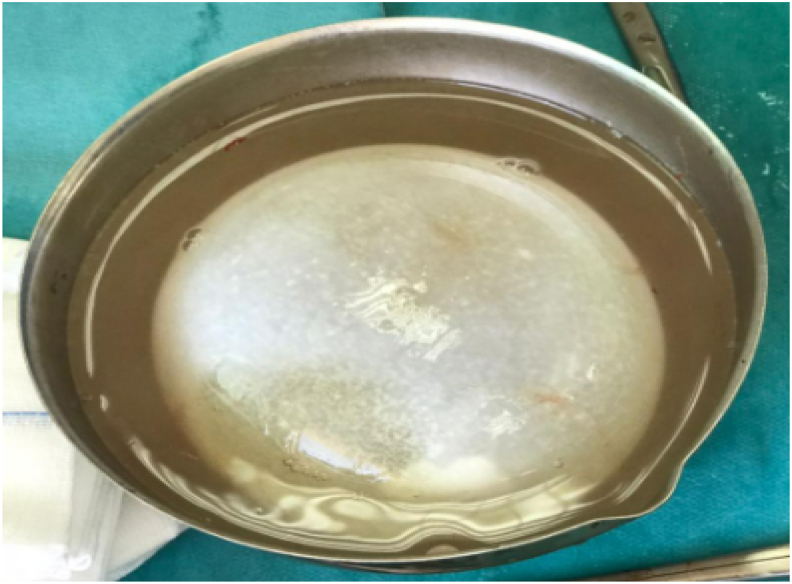
Image showing the complete removal of the hydatid cyst without its rupture in the second patient.

**Fig. 8:**
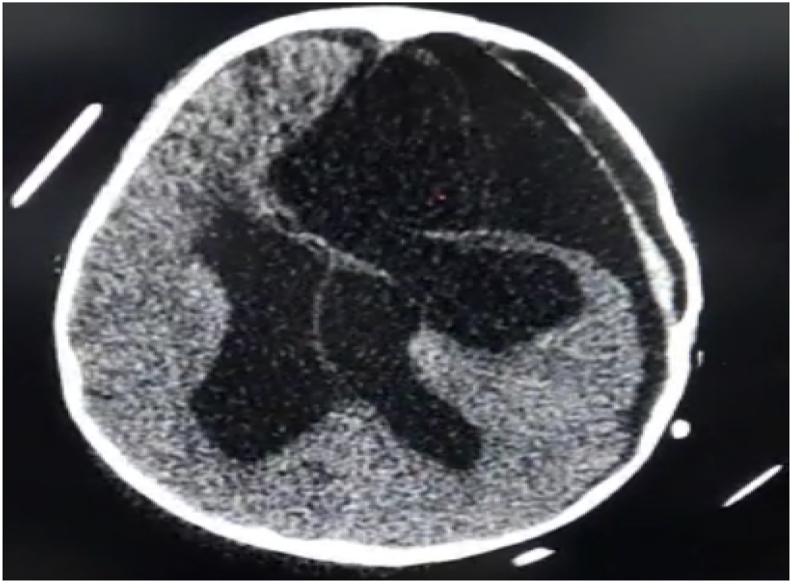
The control CT scan showing the total removal of the hydatid cyst in the second patient.

The intervention was performed by our chief resident under general anesthesia. A large frontoparietal bone flap was performed. The cerebral cortex was laminated with the cyst and gently retracted; a flexible probe was introduced between the parenchyma and the cyst to allow delivery by hydro pulsion according to the Arana-Iniguez technique. Hypertonic saline was then injected. The delivery of the cyst was carried out without incident. Given the wide opening of the dura for the delivery of the cyst, the closure must be tight and careful from the most sloping point to ensure a good filling of the cavity and expel the air and avoid pneumocephalus.

The post-operative follow-up was simple with a total regression of the sensory-motor deficit. Anatomopathological examination confirmed the diagnosis. The patient was put under anti-parasitic treatment based on Albendazole, and under anti-convulsant treatment (LAMOTRIGINE). The patient was discharged 15 days after the surgery.

### 2.2 Second case

5 years old girl with no pathological history and notion of contact with stray dogs living in rural environment. The history of the disease goes back to 8 months by the occurrence of a heaviness of the hemi corps without notions of convulsive seizures or fever and conservation of the general state.

The clinical examination revealed a 3/5 right hemiparesis and left central facial paralysis without any other associated signs. The rest of the somatic examination was unremarkable.

The diagnosis of hydatid cyst was evoked and an extension workup in search of another localization was performed, made of a chest X-ray and an abdominal ultrasound which did not reveal any anomaly.

The intervention took place in the same way as the 1st case according to the Arana-Iniguez technique, done by our professor.

The evolution was marked by the improvement of the sensory-motor deficit. The patient was also put on anti-parasite and anti-convulsant treatment. She was discharged from the hospital after stabilization of her clinical condition and good postoperative evolution in post-op a stay in intensive care of 24 hours or 48 hours depending on the evolution of the patient is necessary. A gentle awakening is advised. After extubating the patient and his return to his place, it is advised to turn the head on the side contralateral to the cyst to avoid the brutal mobilization of the brain on the side of the porencephalic cavity under the effect of gravity while taking care that the neck of the patient is mobilized to avoid a torticollis.

This case has been reported in line with the 2020 SCARE guidelines [2].

## 3 Discussion

Primary intracranial hydatid cyst is generally solitary, whereas secondary cysts are multiple. The cerebral hydatid cyst is secondary to the development of the Echinococcus Granulosis parasite in the intracranial. Normally the dog is the definitive host, but the cycle can be broken, and man can be an accidental host [3].

This localization is rare, the clinical picture is varied according to the localization at the cerebral level associated with a syndrome of intracranial hypertension with or without signs of localization (convulsive seizures, visual disorders, damage to the cranial pairs …)

The neurological examination is very polymorphic and can evolve from an asymptomatic picture to coma (behavioral disorder, motor deficit, cranial pair damage, consciousness disorder) [1,4]. The cranial CT scan shows a rounded, well-limited image with a fluid density close to that of the CSF, homogeneous without peripheral edema [[5], [6], [7]]. The hydatid cyst may take on contrast in a peripheral ring with perilesional oedema when it is infected [[8], [9], [10]].

We can evoke as differential diagnosis on the cerebral scanner with hydatid cyst, a cystic astrocytoma, or a cerebral abscess, but the signs which are not in favor are the absence of perilesional edema and mural nodule, as well as by the absence of contrast enhancement on the walls of the cyst [11]. some images can be pathognomonic like the visualization of floating membrane as well as calcifications even if the latter remains rather rare, less than 1% [12].

Cerebral MRI is the key examination in this pathology, objectifying a hypo-signal T1, hyper-signal T2 image. This examination is the best placed to detect multiple cerebral hydatid cysts and better highlights the anatomical relationship of the lesion with respect to adjacent structures, which allows good surgical planning, while the CT scan is more effective in terms of calcifications [12].

Hydatid serology in cerebral localization is of very low value compared to other localizations. It could be useful for diagnosis and postoperative follow-up despite its low sensitivity. Anatomopathological examination is diagnostic, it highlights the germinal membranes of the cyst [12].

Cerebral hydatid cysts require medical and surgical management. Surgical treatment should be considered whenever possible. The delivery of the cyst by the “Arana-Iniguez” technique is performed by a large bone flap, considering the possibility that the cyst wall adheres to the dura mater. The laminated cortex is separated from the cyst by injecting physiological serum and absorbent cotton. The corticotomy must be equal to 3/4 of the diameter of the cyst. A flexible catheter is introduced between the cyst and the brain. the injection of hypertonic saline through the catheter allows the delivery of the cyst by hydro pulsion [1].

Therefore, the cyst is delivered by the force of the hydro pulsion and gravity, aided by the proper tilt of the head. This technique is easy to implement and allows delivery of the cyst without its rupture and with a minimum of damage to the brain parenchyma. The second possible technique is puncture-aspiration; it is less frequently used and is reserved for cysts at high risk of rupture such as cysts in the fourth ventricle, brainstem, and thalamus [12].

Mebendazole was first used for the treatment of hydatid cyst. However, its absorption from the gut was poor [[13], [14], [15]] so it was soon replaced with albendazole which is more readily absorbed. This drug had a better efficacy because of its metabolite, albendazole sulfoxide, which diffuses easily from cyst membrane and concentrate in the cyst fluid [15,16].

Antiparasitic treatment (Albendazole) is systematic at 15 mg/kg/day for 3 months [17,18]. The medical treatment which has been used by some teams in cases of recurrent, hydatid dissemination, considered inoperable or ruptured intraoperatively, the results of drug treatment of hydatid cysts remain variable depending on the series, with response rates ranging from 43.5 to 80% [11].

Nevertheless, it has some adverse effects including nausea, vomiting, abdominal pain, diarrhea, dizziness, headache and gastro-intestinal disturbances.

according to a study of Shams-Ul-Bari et al., 72 cases of hydatid cyst were randomized into two equal groups. In the first group, patients were subjected to surgery, while patients in the second group were treated with albendazole for 12 weeks before operation and 12 weeks after the operation. None of the patients who received albendazole therapy had viable cysts at the time of surgery while 94.45% of the patients who did not receive albendazole had viable cysts. In patients who did receive albendazole therapy, no recurrence was seen, while among patients who did not receive albendazole, the recurrence rate was 16.66% [16]. Also, it has been shown that systemic use of albendazole before and after surgery reduces the rate of hydatid cyst recurrence. In another study, Saimot et al. treated hydatid cyst patients with albendazole. They showed that the drug was effective in eliminating the cysts and making the operation possible [16].

Its prescription was made essentially according to two protocols: the first with recurrent 1-month courses at 10 mg/kg/day once a day and 15 days of interruption between courses, the second with a continuous 3-month course at 10–12 mg/kg/day twice a day [8,9]. The second protocol, approved by the WHO [9], seems more efficient than intermittent courses, which would be more profitable to the parasite than to the host.

The prognosis is generally good when the diagnosis is made early, allowing early treatment to avoid neurological sequelae. Prevention is ensured by controlled slaughter of cattle [12].

## 4 Conclusion

Cerebral hydatid cyst is a rare condition, affecting mainly children. The diagnosis of cerebral hydatid cyst must be evoked in endemic countries in front of a symptomatology of intra-cranial hypertension. The diagnosis is easily made by CT scan. The treatment is medical-surgical and the prognosis is generally good.

## Ethical approval

Written informed consent was obtained from the parent's patients for publication of this case report and accompanying images. A copy of the written consent is available for review by the Editor-in-Chief of this journal on request.

## Financial disclosure

The authors declared that this study has received no financial support.

## Author contribution

Saad HMADA: Corresponding author writing the paper. Tarek MESBAHI: study concept. Abdelhamid JEHRI: study concept. Abla JOUIDA: study concept. Abdessamad NAJA: Correcting the paper. Naima AMENZAOUI: Correcting the paper. Abdelhakim LAKHDAR: Correcting the paper.

## Provenance and peer review

Provenance and peer review Not commissioned, externally peer-reviewed.

## Please state whether ethical approval was given, by whom and the relevant Judgement's reference number

Written informed consent for publication of their clinical details and/or clinical images was obtained from the patient.

Ethical approval has been exempted by our institution.

## Please state any sources of funding for your research

None.

## Please state any conflicts of interest

The authors declare having no conflicts of interest for this article.

## Research registration unique identifying number (UIN)

ResearchregistryXXXX.

## If you are submitting an RCT, please state the trial registry number – ISRCTN

None.

## Guarantor

Saad HMADA.

## Annals of medicine and surgery author disclosure form

The following additional information is required for submission. Please note that failure to respond to these questions/statements will mean your submission will be returned. If you have nothing to declare in any of these categories then this should be stated.

## Declaration of competing interest

The authors of this article have no conflict or competing interests. All of the authors approved the final version of the manuscript.
